# Fusagerins A–F, New Alkaloids from the Fungus *Fusarium* sp.

**DOI:** 10.1007/s13659-015-0067-1

**Published:** 2015-09-02

**Authors:** Hao Wen, Yan Li, Xingzhong Liu, Wencai Ye, Xinsheng Yao, Yongsheng Che

**Affiliations:** Institute of Traditional Chinese Medicine & Natural Products, College of Pharmacy, Jinan University, Guangzhou, 510632 People’s Republic of China; State Key Laboratory of Toxicology & Medical Countermeasures, Beijing Institute of Pharmacology & Toxicology, Beijing, 100850 People’s Republic of China; State Key Laboratory of Mycology, Institute of Microbiology, Chinese Academy of Sciences, Beijing, 100190 People’s Republic of China

**Keywords:** *Fusarium* sp., Alkaloids, Structure elucidation, Configuration determination

## Abstract

**Abstract:**

Fusagerins A–F (**1**–**6**), six new alkaloids including a unique one with the rare a-(*N*-formyl)carboxamide moiety (**1**), a hydantoin (imidazolidin-2,4-dione) derivative (**2**), and four fungerin analogues (**3**–**6**), were isolated from the crude extract of the fungus *Fusarium* sp., together with the known compound fungerin (**7**). Compound **2** was isolated as a racemate and further separated into two enantiomers on a chiral HPLC column. The structures of **1**–**6** were determined mainly by NMR experiments, and the absolute configuration of **1** and **2** was assigned by electronic circular dichroism (ECD) calculations. Compound **7** showed antibacterial activity against *Staphylococcus aureus* and *Streptococcus pneumoniae*, and weak cytotoxicity against the T24 cells.

**Graphical Abstract:**

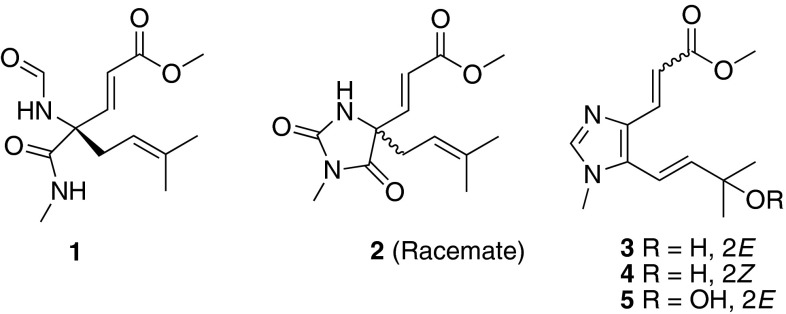

**Electronic supplementary material:**

The online version of this article (doi:10.1007/s13659-015-0067-1) contains supplementary material, which is available to authorized users.

## Introduction

**Fig. 1:**
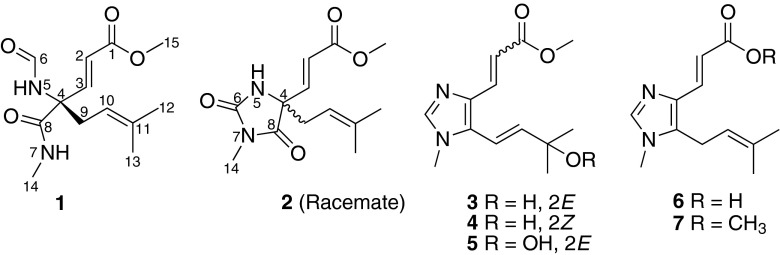
Structures of metabolites **1**–**7** isolated from *Fusarium* sp.

Fungi are important sources of bioactive secondary metabolites [1], and those from special and competitive habitats are especially likely to produce structurally diverse and unique natural products due to their highly evolved secondary metabolism [2–4]. Based on this consideration, and our previous success in the discovery of new bioactive secondary metabolites from the of unique niches [5], a special group of fungi which were isolated from the fruiting body and larvae of *Cordyceps sinensis* were chemically investigated, leading to the isolation of a variety of cytotoxic natural products [6–9]. During the course, we also screened the fungal species isolated from the soil samples that were collected on the Qinghai-Tibetan plateau at altitudes above 3200 m, the environment in which *Cordyceps sinensis* typically reside [10–13]. A strain of *Fusarium* sp. was such a fungus, and an EtOAc extract prepared from solid-substrate fermentation products of the fungus was subjected to chemical investigation. Fractionation of the crude extract afforded fusagerins A–F (Fig. 1; **1**–**6**), six new alkaloids including a unique one with the rare a-(*N*-formyl)carboxamide moiety (**1**), a hydantoin (imidazolidin-2,4-dione) derivative (**2**), and four fungerin analogues (**3**–**6**), along with the known compound fungerin (Fig. 1; **7**) [14]. Details of the isolation, structure elucidation, and biological activities of these compounds are reported herein.

## Results and Discussion

The molecular formula of fusagerin A (**1**) was established as C_13_H_20_N_2_O_4_ (five degrees of unsaturation) on the basis of HRESIMS (*m/z* 269.1497 [M + H]^+^, Δ – 0.1 mmu) and NMR data (Table 1). The ^1^H and ^13^C NMR spectra of **1** showed resonances for two exchangeable protons (*δ*_H_ 7.64 and 7.34, respectively), one aldehyde proton (*δ*_H_ 8.19), four methyl groups including one *O*-methyl (*δ*_H_ 3.71) and one *N*-methyl (*δ*_H_ 2.75), one methylene unit, four olefinic carbons (three of which are protonated), one *sp*^3^ quaternary carbon, and three carboxylic carbons (*δ*_C_ 160.9, 166.8, and 170.6, respectively). These data accounted for all the five unsaturations of **1**. Interpretation of the ^1^H–^1^H COSY NMR data of **1** revealed the presence of three isolated spin-systems, which were C-2–C-3, C-9–C-10, and *N*-7–C-14, respectively. In the HMBC spectrum of **1**, cross peaks from the H-2 and H-3 olefinic protons to the carboxylic carbon at 166.8 ppm (C-1) established an *α,β*-unsaturated ketone moiety (C-1–C-3). An HMBC correlation from the *O*-methyl proton signal H_3_-15 to the C-1 carboxylic carbon located the *O*-methyl group at C-1. Correlations of the H-10 olefinic proton with C-12 and C-13, and of H_3_-12 and H_3_-13 with C-10 and C-11 established of a prenyl unit. In turn, a correlation from H_3_-14 to the C-8 carboxylic carbon revealed the presence of the C-8/*N*-7 amide bond in the structure. In addition, a one-bond coupling was noted between the C-6 carbonyl resonance and the deshielded H-6 proton resonance in the HSQC spectrum, suggesting the presence of formamide functionality. The observation was also supported by a ^1^*J*_C, H_ value of 190 Hz measured in the HMBC spectrum, which was consistent with the reported values of 190 and 189 Hz [15, 16]. Key HMBC correlations from H-2, H-3, H-6, and H-10 to C-4, and from H-3 and H_2_-9 to C-8 indicated that C-3, *N*-5, C-8, and C-9 are all connected to C-4. Although no HMBC correlations were observed for the two exchangeable protons, considering the relevant ^1^H–^1^H COSY correlation between H-7 and H-14, as well as the unsaturation requirement for **1**, they were assigned as 5- and 7-*N*H, respectively. On the basis of these data, the gross structure of fusagerin A was established as shown in Fig. 1.

**Table 1:** NMR data for **1** (600 MHz, acetone-*d*_6_)

Position	*δ*_H_ (*J* in Hz)	*δ*_C_^a^ type	HMBC^b^
1		166.8, qC	
2	5.95, d (15.9)	121.2, CH	1, 3, 4
3	7.20, d (15.9)	148.3, CH	1, 2, 4, 8, 9
4		64.0, qC	
5	7.64, br s		
6	8.19, s	160.9, CH	4
7	7.34, br s		
8		170.6, qC	
9a	2.67, dd (14.5, 7.3)	35.2, CH_2_	3, 4, 8, 10, 11
9b	2.99, dd (14.5, 7.3)		
10	5.04, t, (7.3)	118.1, CH	9, 12, 13
11		136.6, qC	
12	1.69, s	26.1, CH_3_	10, 11, 13
13	1.62, s	18.1, CH_3_	10, 11, 12
14	2.75, d (4.6)	26.7, CH_3_	8
15	3.71, s	51.7, CH_3_	1

^a^Recorded at 150 MHz

^b^HMBC correlations, optimized for 8 Hz, are stated from proton(s) to the indicated carbons

**Fig. 2:**
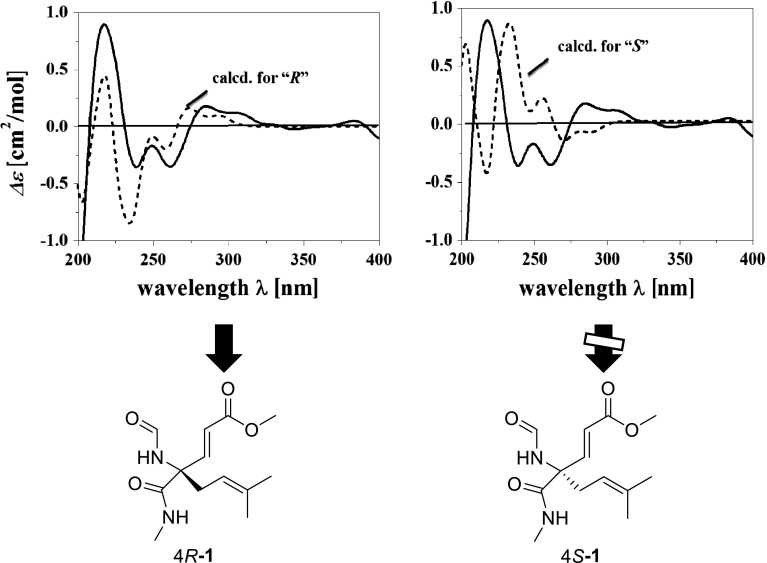
The experimental CD spectrum of **1** (*solid*) and the calculated ECD spectra (*dash*) of two enantiomers 4*R*-**1** and 4*S*-**1**

The C-2/C-3 olefin was assigned the *E*-geometry based on a large coupling constant of 15.9 Hz observed for the corresponding olefinic protons. To determine the absolute configuration for the C-4 stereogenic center in fusagerin A (**1**), the theoretical CD spectra for the two enantiomers 4*R*-**1** and 4*S*-**1** (Fig. 2) were predicted using quantum chemical calculations. Due to the flexible nature of the structure, the conformational species of each enantiomer were defined using the Random Search approach in Sybyl8.0 software. Six conformations were selected from 380 results within a range of 6 kcal/mole above the global minimum. For each conformer, the respective CD spectrum was calculated by TD-B3LYP/6-311++g(2d, p), after optimization using DFT at the B3LYP/6-31G(d) level in Gaussian09 program [17]. The overall calculated CD spectra were then generated according to Boltzmann weighting of each conformer, and were further compared to the experimental CD curve (Fig. 2). The experimental CD spectrum of **1** matched the ECD spectrum for 4*R*-**1**, and was nearly opposite to that for 4*S*-**1**, leading to the deduction of the 4*R* absolute configuration for **1**.

The elemental composition of fusagerin B (**2**) was deduced to be C_13_H_18_N_2_O_4_ (six degrees of unsaturation) by HRESIMS analysis (*m/z* 267.1336 [M + H]^+^, Δ + 0.3 mmu), two mass units less than that of **1**. Analysis of the ^1^H and ^13^C NMR spectroscopic data of **2** (Table 2) revealed the presence of structural features similar to those found in **1**, except that the signals for the H-6 aldehyde proton and the H-7 exchangeable proton in **1** were not observed, and the *N*-methyl proton signal H_3_-14 was observed as a singlet instead of a doublet in the ^1^H NMR spectrum of **2**, implying that the C-6 and C-8 carboxylic carbons are connected to *N*-7 to form a 3-methylimidazolidine-2,4-dione moiety. This postulation was also supported by the HMBC correlations from H_3_-14 to C-6 and C-8. On the basis of these data, the planar structure of **2** was established as depicted (Fig. 1).

**Table 2:** NMR data for **2** (600 MHz, acetone-*d*_6_)

Position	*δ*_H_ (*J* in Hz)	*δ*_C_, type^a^	HMBC^b^
1		166.3, qC	
2	6.13, d (15.8)	122.4, CH	1, 3, 4
3	6.97, d (15.8)	145.8, CH	1, 2, 4, 8, 9
4		66.8, qC	
5	7.57, br s		8
6		156.6, qC	
7			
8		173.5, qC	
9a	2.53, dd (14.5, 7.3)	36.1, CH_2_	3, 4, 8, 10, 11
9b	2.70, dd (14.5, 7.3)		
10	5.05, t (7.3)	116.5, CH	4, 9, 12, 13
11		138.2, qC	
12	1.68, s	26.0, CH_3_	4, 9, 10, 11, 13
13	1.63, s	18.2, CH_3_	4, 9, 10, 11, 12
14	2.89, s	24.6, CH_3_	6, 8
15	3.72, s	52.0, CH_3_	1, 2

^a^Recorded at 150 MHz

^b^HMBC correlations, optimized for 8 Hz, are stated from proton(s) to the indicated carbons

**Fig. 3:**
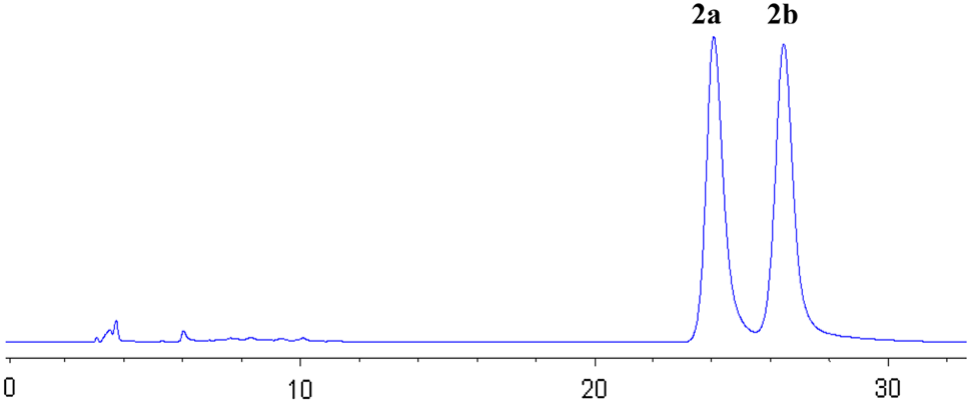
The HPLC chromatogram of **2** using a Kromasil 5-CelluCoat RP column (4.6 × 250 mm, 5 μm) eluted with CH_3_CN/H_2_O (30:70, v/v; flow rate 0.5 mL/min; UV detection at 210 nm)

**Fig. 4:**
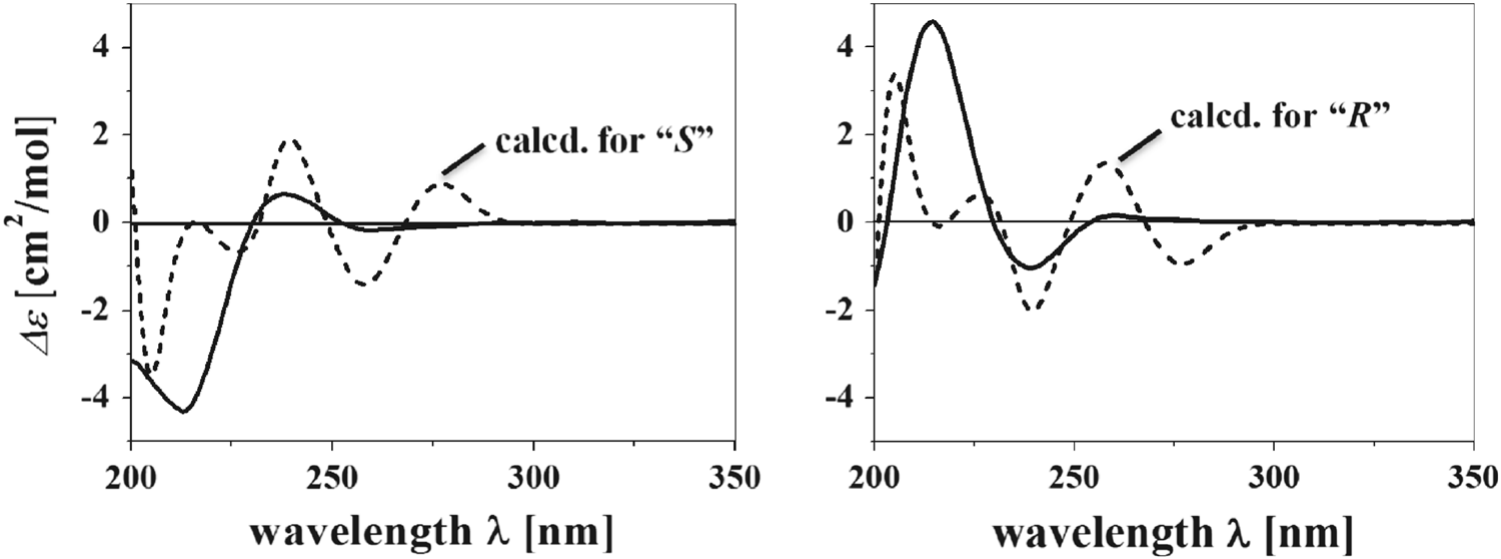
The experimental CD spectrum of **2** (*solid*) and the calculated ECD spectra (*dash*) of two enantiomers, **2a** (4*S*-**2**) and **2b** (4*R*-**2**)

Despite the presence of a stereogenic center at C-4, the measured optical rotation value of fusagerin B (**2**) was zero, and no Cotton effect was observed in its CD spectrum, suggesting that **2** is a racemate. A portion of **2** was separated using HPLC on a chiral column to afford two enantiomers, (−)-fusagerin B (Figs. 3, **2a**) and (+)-fusagerin B (Figs. 3, **2b**), with the measured specific optical rotation values of −16 and +16, respectively (*c* 0.10, MeOH). The absolute configurations of **2a** and **2b** were deduced by comparison of the experimental and simulated ECD spectra for enantiomers 4*R*-**2** and 4*S*-**2** (Fig. 4). The experimental CD spectra of **2a** and **2b** matched the calculated ECD curves of 4*S*-**2** and 4*R*-**2**, respectively, leading to the deduction of 4*S* for (−)-fusagerin B (**2a**), and 4*R* configuration for (+)-fusagerin B (**2b**).

Fusagerin C (**3**) gave a pseudomolecular ion [M + H]^+^ peak at *m/z* 251.1388 (Δ + 0.2 mmu) by HRESIMS, consistent with the molecular formula C_13_H_18_N_2_O_3_ (six degrees of unsaturation). Analysis of its ^1^H and ^13^C NMR data (Table 3) revealed structural similarity to the co-isolated known compound fungerin (**7**) [14], except that the C-11 olefinic carbon in **7** was reduced to an oxygenated *sp*^3^ quaternary carbon (*δ*_C_ 70.8), and the C-9 methylene carbon in **7** was oxidized to an olefinic carbon (*δ*_H_/*δ*_C_ 6.66/112.3), which were confirmed by the ^1^H–^1^H COSY NMR correlations of 9-H with 10-H, and HMBC cross peaks from the exchangeable proton OH-11 to C-10, C-12, and C-13. The C-2/C-3 and C-9/C-10 olefins were all assigned the *E*-geometry based on the large coupling constants observed for the relevant olefinic protons (15.3 and 16.2 Hz, respectively). Therefore, the structure of **3** was determined as shown in Fig. 1.

**Table 3:** NMR data for **3**–**6**

Position	**3**		**4**		**5**		**6**	
	*δ*_H_^a^ (*J* in Hz)	*δ*_C_^b^	*δ*_H_^a^ (*J* in Hz)	*δ*_C_^b^	*δ*_H_^c^ (*J* in Hz)	*δ*_C_^d^	*δ*_H_^e^ (*J* in Hz)	*δ*_C_^f^
1		168.3, qC		168.8, qC		168.4, qC		168.2, qC
2	6.52, d (15.3)	115.1, CH	5.74, d (12.1)	118.2, CH	6.62, d (15.5)	115.9, CH	6.23, d (15.2)	114.0, CH
3	7.64, d (15.3)	136.7, CH	6.68, d (12.1)	129.5, CH	7.59, d (15.5)	135.1, CH	7.47, d (15.2)	135.3, CH
4		135.8, qC		135.9, qC		135.7, qC		133.0, qC
6	7.56, s	140.6, CH	7.43, s	138.9, CH	7.47, s	139.2, CH	7.61, s	139.1, CH
8		133.4, qC		130.8, qC		131.8, qC		134.1, qC
9	6.66, d (16.2)	112.3, CH	6.57, d (16.1)	112.8, CH	6.44, d (16.6)	114.8, CH	3.43, d (7.0)	21.6, CH_2_
10	6.28, d (16.2)	146.2, CH	6.16, d (16.1)	144.8, CH	6.14, d (16.6)	140.9, CH	5.05, t (7.0)	120.0, CH
11		70.9, qC		70.8, qC		82.1, qC		133.0, qC
12	1.40, s	30.3, CH_3_	1.37, s	30.4, CH_3_	1.52, s	24.4, CH_3_	1.68, s	25.3, CH_3_
13	1.40, s	30.3, CH_3_	1.37, s	30.4, CH_3_	1.52, s	24.4, CH_3_	1.73, s	17.7, CH_3_
14	3.70, s	32.7, CH_3_	3.65, s	32.3, CH_3_	3.65, s	32.6, CH_3_	3.53, s	31.2, CH_3_
15	3.70, s	51.4, CH_3_	3.63, s	51.1, CH_3_	3.80, s	51.5, CH_3_		
OH-1							12.07, s	
OH-11^g^	4.89, s		4.79, s					
OOH-11^a^					10.41, br s			

^a^Recorded at 500 MHz in acetone-*d*_6_

^b^Recorded at 125 MHz in acetone-*d*_6_

^c^Recorded at 500 MHz in CDCl_3_

^d^Recorded at 125 MHz in CDCl_3_

^e^Recorded at 400 MHz in DMSO-*d*_6_

^f^Recorded at 100 MHz in DMSO-*d*_6_

^g^Recorded at 100 MHz in DMSO-*d*_6_

The molecular formula of fusagerin D (**4**) was determined to be C_13_H_18_N_2_O_3_ on the basis of HRESIMS (*m/z* 251.1391 [M + H]^+^, Δ – 0.1 mmu) and NMR data (Table 3), which is the same as that of **3**, implying isomeric relationship between the two compounds. The ^1^H and ^13^C NMR spectra of **4** showed nearly identical resonances to those of **3**, except that the chemical shifts for the C-2/C-3 olefin (*δ*_H_/*δ*_C_ 6.52/115.1 and 7.64/136.7 in **3**; 5.74/118.2 and 6.68/129.5 in **4**) were significantly different. Interpretation of the 2D NMR data of **4** established the same planar structure as **3**, and a small coupling constant of 12.1 Hz observed between H-2 and H-3 in **4** compared to 15.3 Hz in **3**, indicating that **4** is the 2*Z* isomer of **3**.

Fusagerin E (**5**) was assigned a molecular formula of C_13_H_18_N_2_O_4_ (six degrees of unsaturation) by HRESIMS (*m/z* 267.1339 [M + H]^+^, Δ 0 mmu), containing one more oxygen atom than that of **3**. Analysis of its NMR data (Table 3) revealed structural characteristics similar to those of **3**, but the ^13^C NMR chemical shift of C-11 in **5** (*δ*_C_ 82.1) was significantly downfield compared to that in **3** (*δ*_C_ 70.9), suggesting that the C-11 hydroxy group was oxidized to a peroxy unit. A similar peroxy moiety was also found in the known compounds, peroxylippidulcine A [18] and bruguierin C [19]. The absolute configuration of **5** was deduced as shown by analogy to **3** and **4**.

The molecular formula of fusagerin F (**6**) was established as C_12_H_16_N_2_O_2_ (six degrees of unsaturation) on the basis of HRESIMS (*m/z* 221.1293 [M + H]^+^, Δ – 0.8 mmu), which is 14 mass units less than that of the known compound fungerin (**7**) [14]. The NMR data of **6** (Table 3) are nearly identical to those of **7**, except that the C-15 methyl group (*δ*_H_/*δ*_C_ 3.77/51.4) in **7** was replaced by a proton (*δ*_H_ 12.07), indicating that **6** bears a free carboxylic acid moiety at C-2.

Compounds **1**–**7** were tested for their antimicrobial activities against a panel of bacteria and fungi [20, 21], Compounds **1**–**6** did not show noticeable in vitro antibacterial or antifungal activities against the above-mentioned organisms (Experimental Section; IC_50_ > 50 μM). Only fungerin (**7**) showed weak antibacterial activity against *Staphylococcus aureus* (ATCC 6538) and *Streptococcus pneumoniae* (CGMCC 1.1692), with IC_50_ values of 33.8 and 34.5 µM, respectively, while the positive control ampicillin showed an IC_50_ value of 0.26 µM. Compounds **1**–**7** were also tested for cytotoxicity against a panel of six human tumor cell lines, HeLa (cervical epithelial cells), A549 (lung carcinoma epithelial cells), MCF-7 (breast cancer cells), HCT116 (colon cancer cells), SW480 (colon cancer cells), and T24 (bladder cancer cells). Compounds **1**–**6** did not show detectable cytotoxicity against the six cell lines at a concentration of 20 µg/mL, whereas compound **7** showed weak cytotoxic effect on the T24 cells, with an IC_50_ value of 42.2 µM (the positive control cisplatin showed an IC_50_ value of 6.71 µM.

**Scheme 1:**
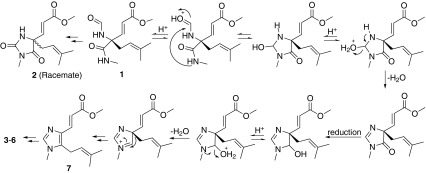
Hypothetical biosynthetic pathways for compounds **1**–**6**

Fusagerin A (**1**) is the first example of naturally occurring *α*-(*N*-formyl)carboxamide, which was only synthesized in situ previously from *α*-aminonitriles using formic-acetic anhydride [22]. Fusagerin B (**2**) is a new member of the extensively studied hydantoin (2,4-imidazolidindione) class of natural products which possess a variety of pharmacological properties, such as anticonvulsant [23–26], antitumor [27, 28], anti-HIV [29], antiarrhytmic [30], antihypertensive [31], hypolipidemic [32], antituberculosis [33], and antifungal activities [34]. However, **2** differs from the known precedents by having different substituents on the 2,4-imidazolidindione ring. The only two fungal metabolites incorporating the hydantoin moiety were both isolated from marine-derived fungi [35, 36]. Other examples of this class of natural products were mainly isolated from marine organisms including exiguamine A [37], agesamides A and B [38], naamidines and isonaamidines [39–47], parazoanthines A–E [48], and 5-methoxy-5-(4-methoxyphenyl)-3-methyl-2,4-imidazolidinedione [49]. Compounds **3**–**6** are closely related to fungerin (**7**) [14], all possessing the imidazole ring. To our knowledge, only three fungerin derivatives have been reported including visoltricin [50], and hydroxyfungerins A and B [51]. Compounds **3**–**6** differ from the above-mentioned natural products mainly on the identities of the side chains. The plausible biogenetic pathways for compounds **1**–**6** are illustrated in Scheme 1.

## Experimental Section

### General Experimental Procedures

Optical rotations were measured on a polAAr3005 polarimeter, and UV data were recorded on a Shimadzu Biospec-1601 spectrophotometer. The CD spectra were recorded on a JASCO J-815 spectropolarimeter. IR data were recorded using a Nicolet Magna-IR 750 spectrophotometer. ^1^H and ^13^C NMR data were acquired with Varian Mercury-400, -500, and -600 spectrometers using the solvent signals (acetone-*d*_6_: *δ*_H_ 2.05/*δ*_C_ 29.8, 206.1; DMSO-*d*_6_: *δ*_H_ 2.49/*δ*_C_ 39.5; CDCl_3_: *δ*_H_ 7.26/*δ*_C_ 76.7) as references. The HSQC and HMBC experiments were optimized for 145.0 and 8.0 Hz, respectively. ESIMS and HRESIMS data were obtained using an Agilent Accurate-Mass-Q-TOF LC/MS 6520 instrument equipped with an electrospray ionization (ESI) source. The fragmentor and capillary voltages were kept at 125 and 3500 V, respectively. Nitrogen was supplied as the nebulizing and drying gas. The temperature of the drying gas was set at 300 °C. The flow rate of the drying gas and the pressure of the nebulizer were 10 L/min and 10 psi, respectively. All MS experiments were performed in positive ion mode. Full-scan spectra were acquired over a scan range of *m/z* 100–1000 at 1.03 spectra/s. HPLC separations were performed on an Agilent 1260 instrument (Agilent, USA) equipped with a variable wavelength UV detector. Chiral HPLC analysis and separation were performed on a Kromasil 5-CelluCoat RP column (4.6 × 250 mm; 5 μm; AkzoNobel).

### Fungal Material

The culture of *Fusarium* sp. was isolated from a soil sample collected on the Qinghai-Tibetan plateau at an altitude of 3800 m, in May, 2008. The isolate was identified based on morphology and sequence (Genbank Accession No. JQ284030) analysis of the ITS region of the rDNA. The fungal strain was cultured on slants of potato dextrose agar (PDA) at 25 °C for 10 days. Agar plugs were cut into small pieces (about 0.5 × 0.5 × 0.5 cm^3^) under aseptic conditions, 15 pieces were used to inoculate in three Erlenmeyer flasks (250 mL), each containing 50 mL of media (0.4 % glucose, 1 % malt extract, and 0.4 % yeast extract), and the final pH of the media was adjusted to 6.5. After sterilization, three flasks of the inoculated media were incubated at 25 °C on a rotary shaker at 170 rpm for 5 days to prepare the seed culture. Spore inoculum was prepared by suspending the seed culture in sterile, distilled H_2_O to give a final spore/cell suspension of 1 × 10^6^/mL. Fermentation was carried out in eight Fernbach flasks (500 mL), each containing 80 g of rice. Distilled H_2_O (120 mL) was added to each flask, and the contents were soaked overnight before autoclaving at 15 psi for 30 min. After cooling to room temperature, each flask was inoculated with 5.0 mL of the spore inoculum and incubated at 25 °C for 40 days.

### Extraction and Isolation

The fermented material was extracted repeatedly with EtOAc (4 × 1.0 L), and the organic solvent was evaporated to dryness under vacuum to afford the crude extract (50.0 g), which was fractionated by silica gel VLC using petroleum ether–EtOAc–MeOH gradient elution. The fraction (150 mg) eluted with 10:90 petroleum ether–EtOAc was separated by Sephadex LH-20 column chromatography (CC) eluting with MeOH. The resulting subfractions were combined and further purified by semipreparative RP HPLC (Agilent Zorbax SB-C_18_ column; 5 μm; 9.4 × 250 mm; 50 % MeOH in H_2_O for 30 min; 2 mL/min) to afford fusagerins C (**3**; 2.0 mg, *t*_R_ 15.80 min), D (**4**; 4.0 mg, *t*_R_ 16.07 min), and B (**2**; 3.4 mg, *t*_R_ 28.10 min). The fraction (150 mg) eluted with EtOAc was also separated again by Sephadex LH-20 CC eluting with MeOH. The resulting subfractions were combined and further purified by RP HPLC (20 % MeOH in H_2_O for 2 min, followed by 20–100 % over 50 min; 2 mL/min) to afford fusagerins E (**5**; 6.3 mg, *t*_R_ 29.40 min) and A (**1**; 3.0 mg, *t*_R_ 32.69 min). The fraction (120 mg) eluted with 99:1 EtOAc–MeOH was further fractionated by Sephadex LH-20 CC eluting with MeOH. The combined subfractions was purified by RP HPLC (65 % MeOH in H_2_O for 15 min, followed by 65–100 % over 60 min; 2 mL/min) to afford fungerin (**7**; 74.8 mg, *t*_R_ 16.32 min). The (140 mg) fraction eluted with MeOH was separated by Sephadex LH-20 CC eluting with MeOH, and the resulting subfractions were purified by RP HPLC (30 % MeOH in H_2_O for 10 min, followed by 30–100 % over 20 min; 2 mL/min) to afford fusagerin F (**6**; 4.0 mg, *t*_R_ 15.24 min). A portion of compound **2** was separated using a Kromasil 5-CelluCoat RP column (30 % CH_3_CN in H_2_O for 30 min; 0.5 mL/min) to afford (−)-fusagerin B (**2a**; 1.0 mg, *t*_R_ 24.30 min) and (+)-fusagerin B (**2b**; 1.0 mg, *t*_R_ 26.78 min).

#### Fusagerin A (**1**)

Pale yellow oil; $\left[ \alpha \right]_{\text{D}}^{22}$ + 5.6 (*c* 0.2, MeOH); UV (MeOH) *λ*_max_ (log *ε*) 210 (2.07), 217 (1.96), 238 (1.73), 285 (1.28) nm; CD (*c* 3.7 × 10^−3^ M, MeOH) *λ*_max_ (Δ*ε*) 218 (+7.28), 239 (–2.89), 249 (–1.36), 261 (–2.85), 285 (+1.45); IR (neat) *ν*_max_ 3325 (br), 2953, 1723, 1666, 1532, 1438, 1384, 1313, 1277, 1200, 1178 cm^−1^; ^1^H, ^13^C NMR, and HMBC data see Table 1; HRESIMS *m/z* 269.1497 (calcd. for C_13_H_21_N_2_O_4_, 269.1496).

#### Fusagerin B (**2**)

Colorless oil; UV (MeOH) *λ*_max_ (log *ε*) 215 (2.62), 237 (1.99) nm; IR (neat) *ν*_max_ 3311 (br), 2929, 1782, 1721, 1658, 1460, 1395, 1317, 1278, 1199, 1176, 1028 cm^−1^; ^1^H, ^13^C NMR, and HMBC data see Table 2. HRESIMS *m/z* 267.1336 (calcd. for C_13_H_19_N_2_O_4_, 267.1339).

#### (−)-Fusagerin A (**2a**)

Colorless oil; $\left[ \alpha \right]_{\text{D}}^{22}$ − 16.0 (*c* 0.1, MeOH); CD (*c* 3.8 × 10^−3^ M, MeOH) *λ*_max_ (Δ*ε*) 213 (–4.32), 238 (+0.65), 260 (−0.18); UV, IR, ^1^H, ^13^C NMR, HMBC, and HRESIMS data were the same as **2**.

#### (+)-Fusagerin (**2b**)

Colorless oil; $\left[ \alpha \right]_{\text{D}}^{22}$ + 16.0 (*c* 0.1, MeOH); CD (*c* 3.8 × 10^−3^ M, MeOH) *λ*_max_ (Δ*ε*) 216 (+4.58), 239 (–1.05), 260 (+0.15); UV, IR, ^1^H, ^13^C NMR, HMBC, and HRESIMS data were the same as **2**.

#### Fusagerin C (**3**)

Colorless oil; UV (MeOH) *λ*_max_ (log*ε*) 237 (2.37), 308 (2.39) nm; IR (neat) *ν*_max_ 3389 (br), 2972, 1720, 1632, 1509, 1438, 1377, 1302, 1278, 1230, 1197, 1173 cm^−1^; ^1^H and ^13^C NMR data see Table 3; HMBC data (acetone-*d*_6_, 500 MHz) H-2 → C-1, C-3, C-4; H-3 → C-1, C-2, C-4, C-8; H-6 → C-4, C-8; H-9 → C-4, C-8, C-10, C-11, C-12, C-13; H-10 → C-8, C-11, C-12, C-13; H_3_-12 → C-9, C-10, C-11, C-13; H_3_-13 → C-9, C-10, C-11, C-12; H_3_-14 → C-6, C-8; H_3_-15 → C-1; (DMSO-*d*_6_, 600 MHz) OH-11 → C-10, C-11, C-12, C-13; HRESIMS *m/z* 251.1388 (calcd. for C_13_H_19_N_2_O_3_, 251.1390).

#### Fusagerin D (**4**)

Colorless oil; UV (MeOH) *λ*_max_ (log*ε*) 238 (2.56), 298 (2.41) nm; IR (neat) *ν*_max_ 3381 (br), 2972, 1708, 1631, 1511, 1436, 1376, 1302, 1264, 1232, 1196, 1172 cm^−1^; ^1^H and ^13^C NMR data see Table 3; HMBC data (acetone-*d*_6_, 500 MHz) H-2 → C-1, C-3; H-3 → C-1, C-2, C-4, C-8; H-6 → C-4, C-8; H-9 → C-4, C-8, C-10, C-11, C-12, C-13; H-10 → C-8, C-11, C-12, C-13; H_3_-12 → C-9, C-10, C-11, C-13; H_3_-13 → C-9, C-10, C-11, C-12; H_3_-14 → C-6, C-8; H_3_-15 → C-1; (DMSO-*d*_6_, 600 MHz) OH-11 → C-10, C-11, C-12, C-13; HRESIMS *m/z* 251.1391 (calcd. for C_13_H_19_N_2_O_3_, 251.1390).

#### Fusagerin E (**5**)

Pale yellow powder; UV (MeOH) *λ*_max_ (log *ε*) 233 (2.31), 293 (2.41) nm; IR (neat) *ν*_max_ 3270 (br), 2973, 1710, 1634, 1515, 1437, 1379, 1262, 1173, 1044 cm^−1^; ^1^H and ^13^C NMR data see Table 3; HMBC data (acetone-*d*_6_, 500 MHz) H-2 → C-1, C-4; H-3 → C-1, C-2, C-4; H-6 → C-4, C-8; H-9 → C-4, C-10, C-11; H-10 → C-8, C-11, C-12, C-13; H_3_-12 → C-10, C-11, C-13; H_3_-13 → C-10, C-11, C-12; H_3_-14 → C-6, C-8; H_3_-15 → C-1; HRESIMS *m/z* 267.1339 (calcd. for C_13_H_19_N_2_O_4_, 267.1339).

#### Fusagerin F (**6**)

Pale yellow powder; UV (MeOH) *λ*_max_ (log*ε*) 236 (1.91), 292 (2.41) nm; IR (neat) *ν*_max_ 3389 (br), 2923, 1688, 1639, 1518, 1415, 1382, 1306, 1274, 1224, 1196, 1148 cm^−1^; ^1^H and ^13^C NMR data see Table 3; HMBC data (DMSO-*d*_6_, 400 MHz) H-2 → C-1, C-3, C-4; H-3 → C-1, C-2, C-4; H-6 → C-4, C-8, C-14; H-9 → C-4, C-8, C-10, C-11; 10-H → C-8, C-9, C-12, C-13; H_3_-12 → C-10, C-11, C-13; H_3_-13 → C-10, C-11, C-12; H_3_-14 → C-6, C-8; HRESIMS *m/z* 221.1293 (calcd. for C_12_H_17_N_2_O_2_, 221.1285).

#### Fungerin (**7**)

^1^H, ^13^C NMR, and the MS data were consistent with literature values [7].

### Computational Details

Conformational analyses of each enantiomer of **1** and **2** were performed using Random Search method with MMFF94s force field and MMFF94 charges in Sybyl8.0 software. For each conformation of both compounds, TD-B3LYP/6-311++g(2d, p)//B3LYP/6-31G(d) calculations yielded excitations with corresponding oscillator and rotational strength values performed with the program package Gaussian09 [17]. Then the overall CD spectra were summed up following the Boltzmann statistics, according to the respective heats of formation. For a better visualization, the rotational strengths were transformed into Δ*ε* values.

### Antimicrobial and Antifungal Bioassays

Antimicrobial and antifungal bioassays were conducted in triplicate by following the National Center for Clinical Laboratory Standards (NCCLS) recommendations [20, 21]. The bacterial strains, *Staphylococcus aureus* (ATCC 6538), *Streptococcus pneumoniae* (CGMCC 1.1692), and *Escherichia coli* (CGMCC 1.2340) were grown on Mueller–Hinton agar, the yeasts, *Candida albicans* (ATCC 10231) and *Geotrichum candidum* (AS2.498), were grown on Sabouraud dextrose agar, and the fungus, *Aspergillus fumigatus* (ATCC 10894), was grown on potato dextrose agar. Targeted microbes (3–4 colonies) were prepared from broth culture (bacteria: 37 °C for 24 h; fungus: 28 °C for 48 h), and the final spore suspensions of bacteria (in MHB medium), yeasts (in SDB medium), and *Aspergillus fumigatus* (in PDB medium) were 10^6^, 10^5^ cells/mL, and 10^4^ mycelial fragments/mL, respectively. Test samples (10 mg/mL as stock solution in DMSO and serial dilutions) were transferred to 96-well clear plate in triplicate, and the suspension of the test organisms was added to each well achieving a final volume of 200 μL (ampicillin and fluconazole were used as positive controls). After incubation, the absorbance at 595 nm was measured with a microplate reader (TECAN). The inhibition rate was calculated and plotted versus test concentrations to afford the IC_50_, whereas the MIC was defined as the lowest test concentration that completely inhibited the growth of the test organisms.

### MTS Assay [52]

In a 96-well plate, each well was plated with (2–5) × 10^3^ cells (depending on the cell multiplication rate). After cell attachment overnight, the medium was removed, and each well was treated with 100 µL medium containing 0.1 % DMSO, or appropriate concentrations of the test compounds and the positive control cisplatin (100 mM as stock solution of a compound in DMSO and serial dilutions; the test compounds showed good solubility in DMSO and did not precipitate when added to the cells). The plate was incubated for 48 h at 37 °C in a humidified, 5 % CO_2_ atmosphere. Proliferation assessed by adding 20 μL of MTS (Promega) to each well in the dark, followed by a 90 min incubation at 37 °C. The assay plate was read at 490 nm using a microplate reader. The assay was run in triplicate.

## Electronic supplementary material

Supplementary material 1 (DOC 431 kb)
