# Supplementary material for: Fusagerins A–F, New Alkaloids from the Fungus Fusarium sp
Source: Nat Prod Bioprospect. 2015 Sep 2;5(4):195–203. doi: 10.1007/s13659-015-0067-1 (PMC4567992; doi:10.1007/s13659-015-0067-1)
Supplement: Supplementary file 1 — Supplementary material 1 (DOC 431 kb) [file 13659_2015_67_MOESM1_ESM.doc]

**Fusagerins A–F, new alkaloids from the fungus *Fusarium* sp.**

Hao WEN,^a^ Yan LI,^b^ Xingzhong LIU,^c^ Wencai YE,^a^ Xinsheng YAO,^a^,* and Yongsheng CHE^b^,*

^a^Institute of Traditional Chinese Medicine & Natural Products, College of Pharmacy, Jinan University, Guangzhou 510632, People’s Republic of China

^b^State Key Laboratory of Toxicology & Medical Countermeasures, Beijing Institute of

Pharmacology & Toxicology, Beijing 100850, People’s Republic of China

^c^State Key Laboratory of Mycology, Institute of Microbiology, Chinese Academy of Sciences, Beijing 100190, People’s Republic of China

**Contents Page**

1) **Figure S1.**  ^1^H NMR spectrum of fusagerin A (**1**; 600 MHz, acetone-*d*_6_) 2

2) **Figure S2.** ^13^C NMR spectrum of fusagerin A (**1**; 150 MHz, acetone-*d*_6_) 3

3) **Figure S3.** ^1^H NMR spectrum of fusagerin B (**2**; 600 MHz, acetone-*d*_6_) 4

4) **Figure S4.** ^13^C NMR spectrum of fusagerin B (**2**; 150 MHz, acetone-*d*_6_) 5

5) **Figure S5.** ^1^H NMR spectrum of fusagerin C (**3**; 500 MHz, acetone-*d*_6_) 6

6) **Figure S6.** ^1^H NMR spectrum of fusagerin C (**3**; 600 MHz, DMSO-*d*_6_) 7

7) **Figure S7.** ^13^C NMR spectrum of fusagerin C (**3**; 125 MHz, acetone-*d*_6_) 8

8) **Figure S8.** ^1^H NMR spectrum of fusagerin D (**4**; 500 MHz, acetone-*d*_6_) 9

9) **Figure S9.** ^1^H NMR spectrum of fusagerin D (**4**; 600 MHz, DMSO-*d*_6_) 10

10) **Figure S10.** ^13^C NMR spectrum of fusagerin D (**4**; 125 MHz, acetone-*d*_6_) 11

11) **Figure S11.** ^1^H NMR spectrum of fusagerin E (**5**; 500 MHz, CDCl_3_) 12

12) **Figure S12.** ^1^H NMR spectrum of fusagerin E (**5**; 500 MHz, acetone-*d*_6_) 13

13) **Figure S13.** ^13^C NMR spectrum of fusagerin E (**5**; 125 MHz, CDCl_3_) 14

14) **Figure S14.** ^1^H NMR spectrum of fusagerin F (**6**; 400 MHz, DMSO-*d*_6_) 15

15) **Figure S15.** ^13^C NMR spectrum of fusagerin F (**6**; 100 MHz, DMSO-*d*_6_) 16

* To whom correspondence should be addressed. Tel/Fax: +86 10 66932679. E-mail: (Y.C.) cheys@im.ac.cn; (X.Y.) tyaoxs@jnu.edu.cn.

**Figure S1.** ^1^H NMR Spectrum of Fusagerin A (**1**; 600MHz, Acetone-*d*_6_)

**Figure S2.** ^13^C NMR Spectrum of Fusagerin A (**1**; 150 MHz, Acetone-*d*_6_)

**Figure S3.** ^1^H NMR Spectrum of Fusagerin B (**2**; 600MHz, Acetone-*d*_6_)

**Figure S4.** ^13^C NMR Spectrum of Fusagerin B (**2**; 150 MHz, Acetone-*d*_6_)

**Figure S5.** ^1^H NMR Spectrum of Fusagerin C (**3**; 500 MHz, Acetone-*d*_6_)

**Figure S6.** ^1^H NMR Spectrum of Fusagerin C (**3**; 600 MHz, DMSO-*d*_6_)

**Figure S7.** ^13^C NMR Spectrum of Fusagerin C (**3**; 125 MHz, Acetone-*d*_6_)

**Figure S8.** ^1^H NMR Spectrum of Fusagerin D (**4**; 500 MHz, Acetone-*d*_6_)

**Figure S9.** ^1^H NMR Spectrum of Fusagerin D (**4**; 600 MHz, DMSO-*d*_6_)

**Figure S10.** ^13^C NMR Spectrum of Fusagerin D (**4**; 125 MHz, Acetone-*d*_6_)

**Figure S11.** ^1^H NMR Spectrum of Fusagerin E (**5**; 500 MHz, CDCl_3_)

**Figure S12.** ^1^H NMR Spectrum of Fusagerin E (**5**; 500 MHz, Acetone-*d*_6_)

**Figure S13.** ^13^C NMR Spectrum of Fusagerin E (**5**; 125 MHz, CDCl_3_)

**Figure S14.** ^1^H NMR Spectrum of Fusagerin F (**6**; 400 MHz, DMSO-*d*_6_)

**Figure S15.** ^13^C NMR Spectrum of Fusagerin F (**6**; 100 MHz, DMSO-*d*_6_)
